# Outer Balloon Ligation Increases Success Rate of Ischemia-Reperfusion Injury Model in Mice

**DOI:** 10.1371/journal.pone.0167631

**Published:** 2016-12-01

**Authors:** Fengwang Hu, Nana Zhai, Wen Gao, Pei Wu, Yuanyuan Luo, Defeng Pan, Yang Liu, Dongye Li

**Affiliations:** 1 Institute of Cardiovascular Disease, Xuzhou Medical University Xuzhou, Jiangsu, China; 2 Department of Cardiology, the Affiliated Hospital of Xuzhou Medical University, Xuzhou, Jiangsu, China; Indiana University School of Medicine, UNITED STATES

## Abstract

**Background:**

Coronary artery disease is a growing public health problem and a major cause of morbidity and mortality. Experimental animal models provide valuable tools for studying myocardial ischemia reperfusion (I/R) injury *in vivo*.

**Objective:**

The purpose of this study was to describe a new method (outer balloon ligation) to induce myocardial I/R injury in mice.

**Methods:**

Ninety-nine male C57BL/6J mice were randomly divided into three groups: sham group, classic method group (I/R-C) and the new method group (I/R-N). The surgical procedure and recovery time were recorded. The levels of TNF-α, IL-6, cTnT and LDH were detected by ELISA kits. Hematoxylin-eosin staining was applied to assess neutrophil infiltration. Moreover, surgical survival, myocardial infarction areas, and cardiac function measurements were also recorded.

**Results:**

The reperfusion operation time in the I/R-N group were markedly less than the I/R-C group (14.73±2.86 vs. 168.60±33.01 sec, *p* <0.0001). Similarly, the recovery time in I/R-N group was shorter than the I/R-C group (45.39±15.39 vs. 101.70±19.33 min, *p* <0.0001). The levels of TNF-α and IL-6 in I/R-N group were also markedly lower than in I/R-C group (136.5±22.21 vs. 170.5±24.79 pg/ml, *p* <0.05 and 100.3±23.74 vs. 144.40±22.24 pg/ml, *p* <0.001). Compared I/R-N group with I/R-C group, the levels of neutrophil infiltration, cTnT and LDH had no significant differences. Surgical survival rate was 96.7% in the I/R-N group, which was significantly improved compared to the rate of 80% in the I/R-C group. However, there were no significant differences in the areas of myocardial infarction and cardiac function between the two groups.

**Conclusions:**

Compared with the classic method, our new method of inducing myocardial I/R injury has higher efficiency and less tissue damage in mice, but achieves the same modeling effects.

## Introduction

Coronary artery disease (CAD) is a heavy burden on public health and is the leading cause of morbidity and mortality [1]. Although there are several treatments for CAD, including thrombolytic therapy, percutaneous coronary intervention, and coronary artery bypass surgery[2], myocardial ischemia-reperfusion (I/R) injury still plays a vital role in causing damage to the myocardium. Accordingly, how to attenuate myocardial I/R injury continues to be the focus of basic and clinical research.

Animal models of myocardial I/R injury are valuable tools for researching the complex pathophysiologic process of I/R injury [3, 4]. There are many mechanisms involved in the I/R process, such as oxidative stress, calcium overload, mitochondrial dysfunction, and activation of inflammatory factors[5,6]. Currently, there are three primary models of I/R injury: Langendorff (isolated heart perfusion system), isolated working heart (modified on Langendorff) and ligation of left anterior descending branch of coronary artery (LAD occlusion) [7–10]. Among the models, the Langendorff and isolated working heart models are mainly used to study acute global or regional I/R and left ventricular work. These methods are based on an *ex vivo* model which cannot achieve the *in vivo* experimental requirements. The LAD occlusion model is considered to be the classic *in vivo* model, and is usually applied to study myocardial I/R injury or myocardial infarction.

In the classic method of LAD occlusion, the operation is performed through a left thoracotomy, then the LAD is exposed and occluded using a ligature. After myocardial ischemia, the ligature is loosened to produce the reperfusion event. During the process, ventilation and a double-chest opening often lead to extensive tissue damage, a low survival rate, and is quite time-consuming [11–13]. More importantly, surgical trauma can also trigger inflammatory processes, which are difficult to distinguish from the inflammatory response caused by myocardial I/R injury [3]. Jong WM et al have designed an experiment to study the inflammatory response in open-chest cardiac I/R model. Their data indicated that the major part of the inflammatory responses in the open-chest model was not due to myocardial I/R injury, but could be ascribed to surgical stressors. And surgical stressors might contribute approximately 75% of the inflammatory response to open-chest cardiac I/R [14]. Such disadvantages are the basis for improving the existing myocardial I/R injury model.

Balloon catheters have a long history in experimental and clinical medicine. Since the very beginning, balloon catheters have been widely utilized with apparent superiority in recent years. Indeed, balloon catheters have been used in animal models of myocardial ischemia [15, 16]. Lukács et al. reviewed studies involving large animal models of myocardial infarction and referred to the technique based on balloon catheter as “balloon occlusion method” [17]. As an improvement to the classic method of LAD occlusion, balloon occlusion LAD is the current mainstream method used in I/R models, especially experiments involving large animals (dogs, swine, sheep, and baboons). For example, Krombach et al. developed a minimally invasive method for creating reperfusion using an inflatable balloon catheter inserted into the LAD in swine [18]. Now, the minimal invasive interventional cardiology technique is named as endovascular balloon occlusion method, it is applied widely in the myocardial infarction model as a closed chest method in vivo. In this method, a balloon catheter was advanced into the LAD to perform I/R by inflation and deflation of balloon. It has better survival rates and less time consumption in comparison with open-chest method.

However, there is considerable difficulty in utilizing the endovascular balloon occlusion LAD method in small animal models, especially in mice, due to the small size of their heart and coronary artery compared with large animals. Therefore, we adopt a modified method of laying the balloon outside of LAD (outer balloon ligation) to induce I/R injury in mice, which produces less tissue damage and operation time, resulting in more efficient myocardial reperfusion.

## Materials and Methods

### Animals and surgical materials

All of the experimental protocols were approved by the Animal Ethics Committee of Xuzhou Medical University, Xuzhou, Jiangsu, China (permit number: CMCACUC2016-01-101), and all procedures were performed with minimal discomfort to the experimental animals. A total of 99 male C57BL/6J mice (8–11 weeks old, weighing 24.88±2.93g) were obtained from the Animal Laboratory of Xuzhou Medical University and randomly divided into three groups: sham group (n = 23), classic method of I/R injury group (I/R-C, n = 38), and new method of I/R injury group (I/R-N, n = 38).

**Fig 1:**
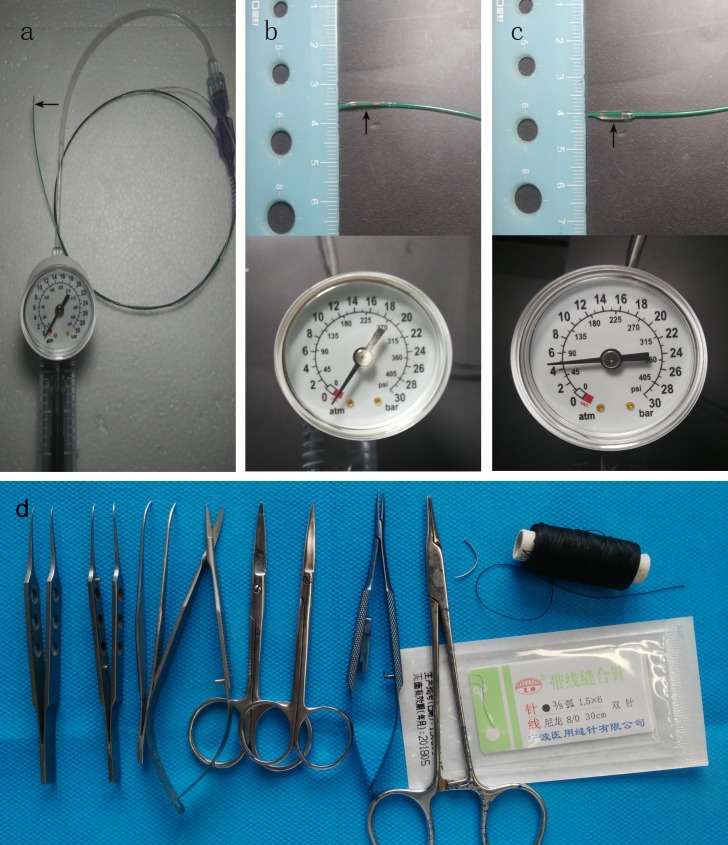
Photographs of the balloon and pressure pump. a. Balloon connected with the pressure pump. b. When the pressure pump was at negative pressure, the balloon was deflated. c. When the pressure pump was at 4–6 atm, the balloon was inflated. Arrows in photographs show the balloon. d. Instruments from the left to the right: smooth tying forceps, toothed tying forceps, curved toothed forceps, Vannas scissors, straight ophthalmic scissors, curved ophthalmic scissors, microneedle holder, needle holder, suture needle,4–0 Non-absorbable surgical suture, surgical suture needles with thread.

The balloon (2.5 × 8 mm; Abbott Vascular Coyol Free Zone, EI Coyol Alajuela, Costa Rica) was connected with a pressure pump (Fig 1A). As shown in Fig 1B, the outer diameter of the deflated balloon was approximately 1 mm when the pressure pump was at negative pressure. When the pressure level of the pump was 4–6 atm, the outer diameter of the inflated balloon with 1–1.5 ml of water was 2.5 mm (Fig 1C).

As the body temperature had critical influence on the outcome and infarction sizes [19, 20], we performed the operative procedure at room temperature of 25°C by air conditioner, a controllable heating pad and a thermometer with a rectal probe were used to maintain and monitor body temperature at 37°C throughout the surgical procedure.

### Anesthesia and ventilation

**Fig 2:**
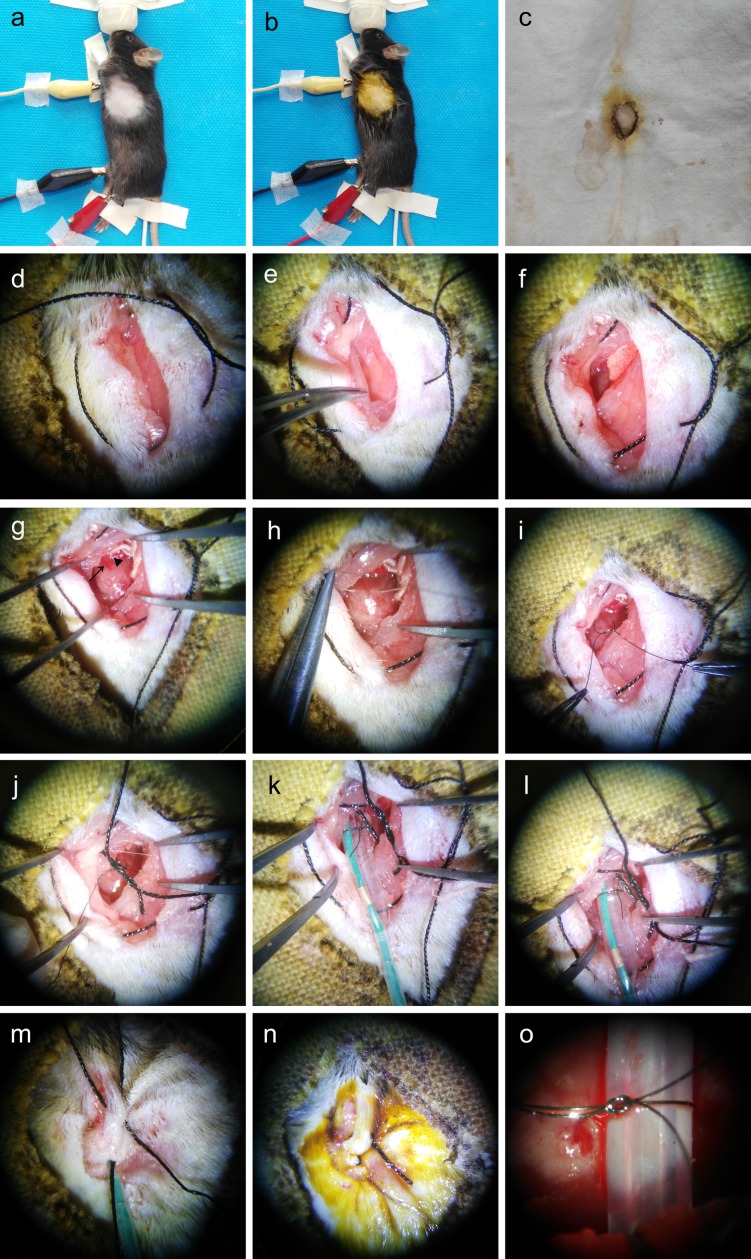
Photographs of various stages of the new method. a, b. The mouse was located on its right side and connected with ventilation and a Data Analysis System. The skin was depilated and disinfected. c. The skin was covered with aseptic hole-towel. d. A 1 cm skin incision and a purse suture were made. e. The pectoralis muscles were separated and the intercostal muscles were exhibited. f. A small hole was made at the fourth intercostal muscle. g. A small wet sterile gauze was used to protect the lungs. The LAD (↑) and left auricle (◀) were displayed. h. One hand held the microneedle holder with a surgical suture needle to pass underneath the LAD and another hand held a smooth tying forceps to widen the hole. i. An approximate 2-mm diameter loop was made. j. A 4–0 suture was passed through the third and fifth intercostal muscle. k. A deflated balloon was placed in the loop. l. The balloon was inflated. m. The chest cavity was closed by straining the purse suture. n. The wound was closed. o. In the classic method, a PE-10 tube was used and a slipknot was made.

All of the animals were anesthetized with an intraperitoneal injection of pentobarbital sodium (100 mg/kg). The absence of toe-pinch reflexes indicated adequate anesthesia. In the present study, all of the mice received once injection of pentobarbital sodium and none of the mice needed an additional bolus of pentobarbital. Mice were given non-invasive ventilation (oral–nasal) with room air (HX-300S, Chengdu Taimeng Technology Corp., Chengdu, China) (Fig 2A and 2B).

### Experimental Protocols

#### New method of I/R injury

In this new method, mice were placed in the right lateral position. When the mice were anesthetized, the fur of the left chest was removed from the skin (Fig 2A). After disinfected with Betadine, the skin was covered with aseptic hole-towel (Fig 2B and 2C). Then, a 1 cm skin incision, which parallels the mid-axillary axial line, was made using straight ophthalmic scissors. After dissecting the subcutaneous tissues, the pectoralis muscles were displayed. A purse suture was then placed using 4–0 suture, as shown in Fig 2D.

After the pectoralis muscles were gently segregated, the intercostal muscles were exhibited (Fig 2E). Then a small hole was made at the fourth intercostal muscle and the heart was exposed (Fig 2F). To avoid injury to the intercostal vessels, the hole was made away from the bottom of the upper rib. In order to protect the lungs intra-operatively, a small wet sterile gauze was used to push the lungs towards the right side (Fig 2G).

The pleural membrane and pericardium were then gently dissected to enhance visualization of the LAD under a dissection microscope (SMZ-168; Motic, Xiamen, Fujian, China), as shown in Fig 2G. The LAD is derived from the aorta and runs underneath the left auricle to the apex of the heart, and is variable in anatomy [21–23]. However, it is sometimes difficult to distinguish the LAD from the vein, especially for inexperienced researchers. The LAD pulses strongly and is bright red. If the LAD is not visible, elevating the left auricle with a smooth tying forceps can display the origin of the LAD.

The site of LAD ligation depends on the volume of infarction designed. However, it is easy to injury the left auricle and cause of high mortality, if the ligation site is too close from its origin. In many studies, the ligate positioning of LAD was 1–2 mm below the tip of the left auricle [22, 24–26]. After the site of ligation has been decided, a surgical suture needle with 8–0 silk suture was passed underneath the LAD (Fig 2H). The depth of the needle was ≤ 1.5 mm because the needle may pierce the left ventricle and lead to bleeding, but too shallow because the needle may injure the LAD. Next, a deflated balloon was placed (Fig 2F) at the ideal site. The suture had a loose double knot, leaving an approximate 2-mm diameter loop to immobilize the balloon (Fig 2I). In this step, a good vision of LAD could help you very much. Thus, in many studies, a rip retractor was used to keep the hole open widely. In the present study, two modifications could keep the hole open without a rip retractor when the needle passed underneath the LAD. Firstly, keep the left upper limb up enough (Fig 2A). Secondly, one hand held the microneedle holder with a surgical suture needle to pass underneath the LAD, then another hand held a smooth tying forceps to widen the hole (Fig 2H). A 4–0 suture was then passed through the third and fifth intercostal muscle to close the wound when the operation was finished (Fig 2J).

Afterwards, a deflated balloon was placed at the site. Finally, the balloon was loosely secured around the LAD (Fig 2K). Ligation of the LAD was accomplished by gently inflating the balloon until an ST segment elevation appeared on the ECG and the left ventricle became pale (Fig 2L). Usually, the pressure of pump was inflated to 2–4 atm so as to induce ischemia. After taking the small wet sterile gauze out, the chest cavity was closed by straining the purse suture (Fig 2M). After 30 min of ischemia, the balloon was deflated and pulled out of the chest carefully associated with LAD reflow. Meanwhile, the ST segment of the ECG declines, which indicated that the myocardium was reperfusing. The operation was completed by closing the wound (Fig 2N).

Approximately 10 min later, the mice could breathe spontaneously. Then, the oral–nasal mask was removed and the mice were placed in a warm cage. During the first 2 hours after surgery, the respiration and consciousness of mouse were observed per 10 min. From the 3rd hour after surgery, the respiration and consciousness were monitored per hour until the end of experiment. At the end of experiment, the mouse was sacrificed with an overdose of pentobarbital sodium (150 mg/kg).

**Fig 3:**
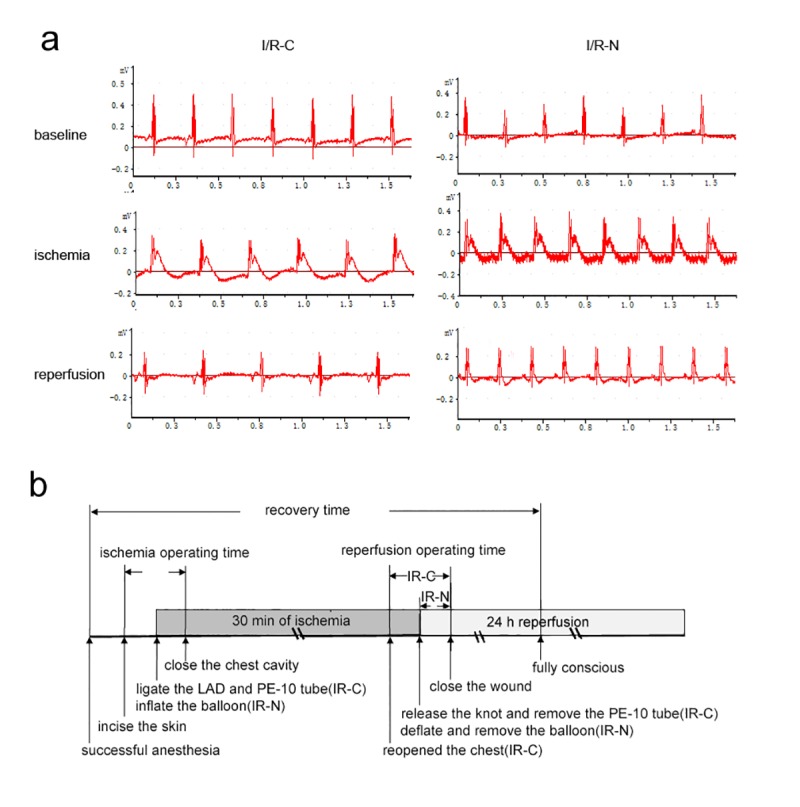
Representative electrocardiogram during different period and a schematic diagram of surgical procedure. a. Electrocardiogram changes during I/R. Before ligation of the LAD, the ST segment was located at the baseline. The ST segment elevated during ischemia and it declined in reperfusion period. b. The schematic diagram of surgical procedure, recovery time, ischemia operating time and reperfusion operating time.

In the present study, the time required for the surgical procedure was referred to as the “surgical procedure time,” and included the ischemia and reperfusion operating times. The ischemia operating time was defined as the interval from beginning the operation to closing the chest cavity. The reperfusion operating time was defined as the time spent to make the LAD reflow. In I/R-N group, the reperfusion operating time was the interval from deflated and pull out the balloon to close the wound. In I/R-C group, the reperfusion operating time was the interval from removed PE-10 tube through reopening chest to close the wound. The recovery time was defined as the time interval from successful anesthesia to fully conscious of mice (Fig 3B).

#### Classic method of I/R injury

In the classic method of I/R injury, the operation was very similar to the new method and the balloon was replaced by a PE-10 tube (Fig 2O). After 30 min of ischemia, the chest was re-opened for slipknot release, then re-closed [24].

#### Sham operation

The procedure used in the sham group was the same as the classic method of I/R injury, except that the suture was passed underneath the LAD but was not ligated.

### Electrocardiogram (ECG) recording

To confirm successful ligation of the LAD, a Data Analysis System (BL-420; TME Technology, Chengdu, Sichuan, China) was used to record the ECG during the I/R period. As shown in Fig 3A, there was a typical elevation of the ST segment when the LAD was occluded, while the ST segment normalized after reperfusion.

### Measurement of plasma levels of TNF-α, IL-6, cTnT and LDH

The plasma levels of TNF-α and IL-6 were measured at 24h post-surgery by using TNF-α and IL-6 ELISA kits (KGEMC102a and KGEMC004, KeyGEN BioTECH, Nanjing, China) following the manufacturer’s instructions. The plasma level of Troponin T (cTnT) and lactate dehydrogenase (LDH) were determined with cTnT and LDH ELISA kits respectively (Cloud-Clone Corp, Houston, USA) at 24h post-surgery.

### Assessment of myocardial polymorphonuclear neutrophil (PMN) infiltration

Mice were subject to 30 min of ischemia and 24 h of reperfusion. The heart was excised and stored in a 10% neutral buffered Formalin solution. After 24h, the midventricular cardiac sections were embedded in paraffin and cut into 4-um slices. The sections were then stained with hematoxylin-eosin. PMNs were counted by a blinded pathologist and presented as per 400× power field of section. Four fields from four independent slices in each animal were observed. The average was used as the degree of PMN infiltration within the ischemic and reperfused area.

### Measurements of myocardial infarct size

The areas of myocardial infarction were measured by staining heart sections with Evans blue and 2,3,5-triphenyltetrazolium chloride (TTC; Sigma, St. Louis, MO, USA). We adopted a method of Gao et al [4]. Briefly, after 24 h of reperfusion, the LAD was tied again and 0.2 ml 2% Evans blue was injected into right ventricle slowly. When the Evans blue was distributed in the heart, the heart was rapidly excised and immersed in 30mM KCl to ensure the heart was arrested in diastole. After 30 min frozen at -80°C, the hearts were cut into 5 slices across the long axis. About 1.2 mm thick per slice. The slices were incubated in 1% TTC for 20 min at room temperature, then incubated in 10% formaldehyde solution for 1 h at room temperature. The infarct area (color in white) could be distinguished from the area at risk (AAR) stained brick red and white, while the area not at risk was stained blue. Slices were digitally photographed and the area at risk and infarct size were determined using Image-Pro Plus, v6.0 (Media Cybernetics, Rockville, MD, USA). The infarct size was presented as the percentage of the area at risk. The area at risk size was presented as the percentage of the total cross-sectional areas of the heart.

### Hemodynamic parameters

Twenty-four hours after surgery, mice were re-anesthetized with 1% isoflurane (v/v). The right carotid artery was exposed for a length of 5–6 mm. Then, a modified PE tube filled with heparin was connected to a Biopac system (BL-420S; Taimeng Technology, Chengdu, China) and advanced into the left ventricle through the right carotid artery. The heart rate (HR), left ventricular systolic pressure (LVSP), left ventricular end-diastolic pressure (LVEDP), rise in ventricular pressure (+dp/dt), and fall in ventricular pressure (-dp/dt) were recorded.

### Statistical analysis

All data in this study are shown as the mean ± Standard Deviation (SD) and a probability (*p*) value of <0.05 was considered statistically significant. Comparisons among all groups were conducted by one-way analysis of variance, followed by the Bonferroni *post hoc* correction test. Kaplan–Meier analysis was used to determine survival. All statistical analyses were performed using GraphPadPrism5 software (GraphPad Software, Inc., La Jolla, CA, USA).

## Results

### The surgical procedure and recovery time for the new method were less than the classic method

**Fig 4:**
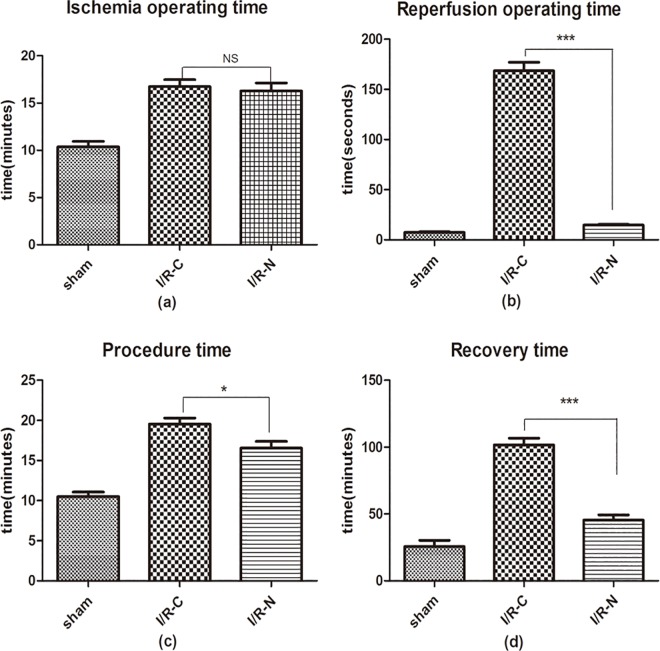
The surgical procedure time, reperfusion operating time and recovery time in new method were less than those of classic method. Ischemia operating time was no difference between two groups. The numbers of mice in sham, I/R-C and I/R-N group were 7, 15, 15 respectively. #*p*<0.05, ### *p*<0.001 vs. sham; *p<0.05, * * *p<0.0001 vs. I/R-C.

As shown in Fig 4, the ischemia operating time was not significantly different between the I/R-N and I/R-C groups (16.30±3.20 vs. 16.74±2.83 min); however the reperfusion operating time in the I/R-N group was shorter than the I/R-C group (14.73±2.86 vs. 168.60±33.01 sec, *p*<0.0001).

To determine the surgical operation damage in the two groups, the recovery time was recorded. The recovery time in the I/R-N group was significantly shorter than the I/R-C group (45.39±15.39 vs. 101.70±19.33 min, *p*<0.001).

### The new method caused less inflammation and equal cardiac damage

**Fig 5:**
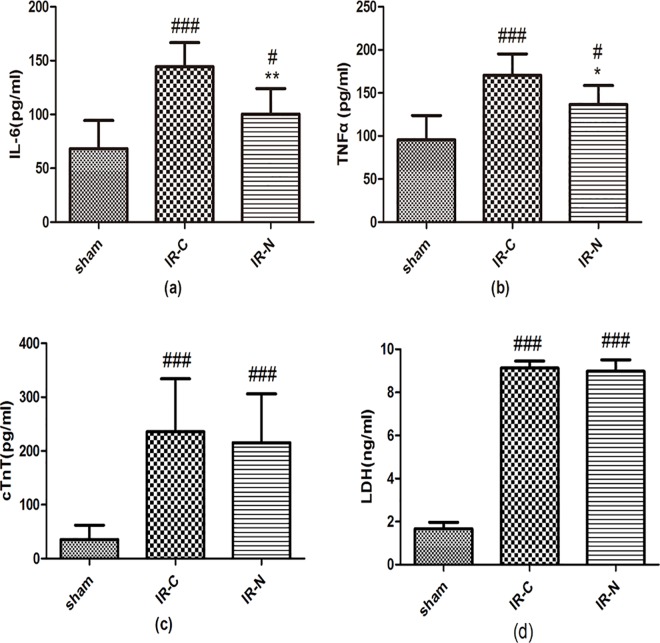
The new method has less inflammation and equal cardiac damage. The plasma concentration of IL-6 (a) (n = 8), and TNF-α (b) (n = 8), cTnT (c) (n = 8) and LDH (d) (n = 4) were measured at 24 h after surgery in the different groups. The plasma levels of TNF-α and IL-6 in I/R-N group was significantly lower than I/R-C group. However, the plasma levels of cTnT and LDH were no difference between I/R-N group and I/R-C group. #*p*<0.05, ###*p*<0.001 vs. sham;*p<0.05, * *p<0.01 vs. I/R-C.

To evaluate the inflammatory reaction occurring 24 h after surgery, the plasma levels of TNF-α and IL-6 were measured. As shown in Fig 5, the IL-6 level in the I/R-N group was significantly lower than the I/R-C group (100.3±23.74 vs. 144.40±22.24 pg/ml, *p*<0.001). The IL-6 level in both groups was increased compared with the sham group (68.25±26.22 pg/ml). Similarly, the TNF-α level was decreased in the I/R-N group compared with the I/R-C group (136.5±22.21 vs. 170.5±24.79 pg/ml, *p*<0.05). The TNF-α levels in both groups were higher than the sham group (95.60±28.26 pg/ml) (Fig 5A and 5B).

The plasma levels of troponin T, which reflected degree of cardiac specific damage, were measured at 24 h after surgery. The levels of cTnT were significantly elevated in I/R-N group and I/R-C group following I/R (215.50±90.68 pg/ml and 235.80±98.25 pg/ml, p>0.05) compared to sham group (35.47±26.85 pg/ml, both p<0.001). Similarly, the LDH release was not significantly different between I/R-N group and I/R-C group (8.97±0.19 vs. 9.13±0.12 ng/ml, p>0.05). The LDH release was significantly increased in I/R-C and I/R-N groups compared with sham group (1.67±0.11 ng/ml, both p<0.001) (Fig 5C and 5D).

### Neutrophil accumulation in the myocardium

**Fig 6:**
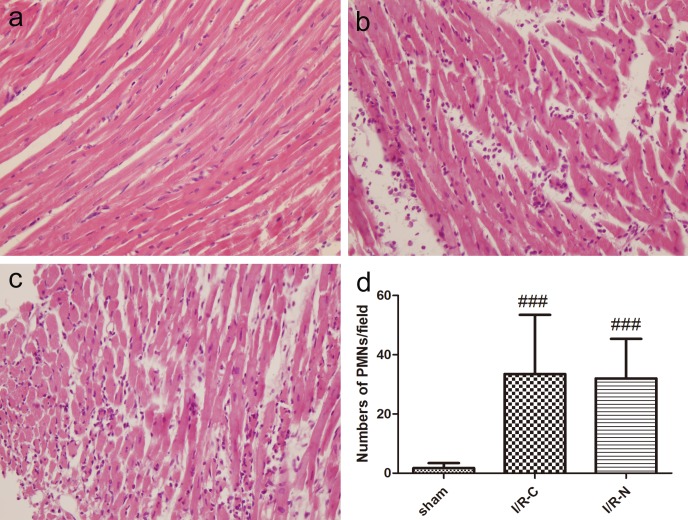
Neutrophil infiltration analysis. a-c. Representative hematoxylin-eosin staining myocardium images in sham group, I/R-C group and I/R-N group respectively (400×). Myocardium injury was serious within the ischemic areas of the heart after I/R. d. Neutrophil accumulation within the ischemic areas of the heart after I/R. Large number of neutrophil were accumulate in the cardiac ischemic areas after I/R (n = 4). ###*p*<0.001 vs sham group.

The hematoxylin-eosin stained images of myocardium and infiltration of PMNs into myocardium were presented in Fig 6. The number of PMNs was significantly increased in I/R-C and I/R-N groups compared with sham group, and there were no significant differences between I/R-N group and I/R-C group (32.00±13.38 vs. 33.50±19.99 /field).

### The new method improved the surgical survival rate

**Fig 7:**
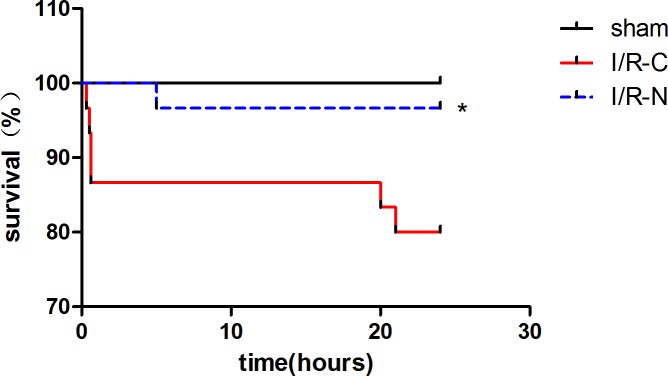
The new method improved surgical survival. Survival rates at 24 h in sham (n = 15), I/R-C (n = 30), and I/R-N groups (n = 30). *p<0.05 I/R-N vs. I/R-C.

One mouse died (3.3%; Fig 7) due to a hemothorax in the I/R-N group after surgery. In the I/R-C group, 6 mice died (20%; Fig 7); 4 mice died from intra-operative arrhythmias and 2 mice died of unknown reasons post-operatively.

### Ischemia reperfusion induced myocardial injury and decreased cardiac function

**Fig 8:**
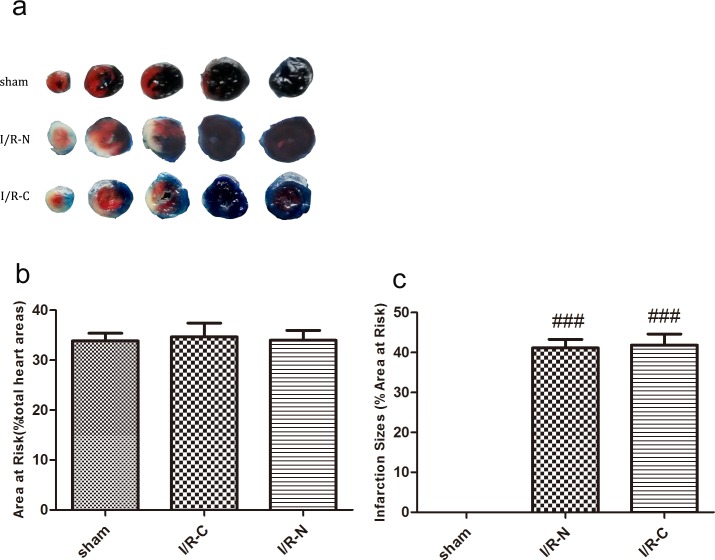
Myocardial injury in different groups. a. Representative photographs of Evans blue/TTC-stained heart sections in different groups. b. Percentage of area at risk over total cross-sectional areas of the heart in different groups (n = 6). c. Percentage of infarct size over area at risk in different groups (n = 6). ###*p*<0.001 vs. sham group.

Evans blue/TTC double staining was used to stain the myocardial infarction areas. The left ventricular infarction size was presented as the percentage of infarct area over area at risk. AAR was presented as the percentage of area at risk over total heart area. As shown in Fig 8, the left ventricular infarction size was not significantly different between the I/R-N and I/R-C groups (41.15±2.13% vs. 41.85±2.77%, *p*>0.05). Similarly, the area at risk was not significantly different between the I/R-N and I/R-C groups (33.98±1.97% vs. 34.63±2.77%, *p*>0.05) (Fig 8).

Twenty-four hours after reperfusion, the hemodynamic indices showed that LVSP, +dp/dt, and -dp/dt were significantly decreased and LVEDP was increased in both I/R groups compared with the sham group. The heart rates were not significantly different between the I/R and sham groups (Table 1).

**Table 1:** Hemodynamic parameters in different groups.

Parameters	Sham(n = 4)	I/R-C(n = 4)	I/R-N(n = 4)
Heart rate (beats/min)	429.0±33.74	453.3±13.52	441.50±13.03
LVSP (mmHg)	105.60±13.52	95.41±1.64**	93.99±1.98**
LVEDP(mmHg)	3.13±3.07	12.88±0.86***	13.97±1.13***
+dp/dt(mmHg/s)	3892±180.90	2956±128.70***	2823±162.00***
-dp/dt(mmHg/s)	-3780±75.60	-2775±152.90***	-2638±187.90***

Values are means ± SD; n, number of animals. LVSP, left ventricular systolic pressure; LVEDP, left ventricular end-diastolic pressure; +dp/dt, rise of ventricular pressure; -dp/dt, fall of ventricular pressure.

**p<0.01

***p<0.001 vs. sham group.

## Discussion

Experimental animal models *in vivo* are vital to study human disease. With respect to CAD, experimental animal models provide us valuable tools for studying myocardial I/R. Many species have been utilized to make myocardial I/R models, including large mammals and small mammals [27]. Although large mammals are more similar to humans than small mammals in terms of cardiovascular physiology and pathology, the efficiency ratio of large mammal myocardial I/R models is low. In contrast, the myocardial I/R models of small mammals are simple and practicable, researchers currently tend to choose small mammals as I/R experimental animals models, rats and mice are preferred. Thus, researchers prefer to choose mice instead of other animals as an experimental animal model. Currently, it has been accepted that the murine myocardial I/R model is an effective method for cardiovascular research simulating clinical acute or chronic heart disease [3,4]. Therefore, mice were chosen as the experimental animal model in the present study.

Several laboratories have introduced various methods to establish murine myocardial I/R models [28, 29]. Among these methods, the classic method has been widely accepted and applied [29]. In addition, this method can induce myocardial I/R using a standard approach; however, during the operation period, the chest cavity must be re-opened to loosen the slipknot to perform reperfusion. This procedure could add surgical injury along with increased operation time and difficulty. An improved method was implemented by Gao et al, who placed the heart outside the chest, ligated the LAD artery, then replaced the heart inside the chest [28]. Reperfusion was achieved by pulling the long silk out of the chest. The improvement reduced the procedure time, and avoided the use of a ventilator; however, this improved method required experienced and skilled technicians, and thus cannot be widely applied. To overcome these drawbacks, a novel and efficient surgical procedure has been developed for inducing myocardial I/R injury mice in the current study.

In our study, the balloon was used to induce myocardial I/R injury in mice. The coronary I/R course was set by the inflation and deflation of the balloon. The balloon method is usually applied in myocardial I/R and infarction models [30–32]. First, the balloon was used to induce the myocardial infarction model by transluminal balloon occlusion in swine [30]. In view of the small caliber of coronary arteries in small mammals, it is difficult and unlikely to place the balloon into the coronary artery. The myocardial I/R and infarction model of small mammals, such as rats and mice, can be established using the open chest method only. To improve balloon application, an inflatable balloon secured around the left coronary artery was used to induce the rat myocardial infarction model [31, 32]. The balloon ligation applied in the I/R model of rats has piqued our interest. Therefore, we have tried, for the first time, to use outer balloon ligation to establish a myocardial I/R model in mice. This procedure improved the success rate of surgery, reduced surgical injury, and shortened the operative time. Through the use of a balloon, our new method involved opening the chest cavity once, which was also a benefit for lung recruitment.

The new approach for inducing an I/R model has four advantages compared with the classic approach. The new method had less reperfusion operation time, tissue damage, and inflammation reaction, and faster recovery and higher surgical survival than the old method. Of equal importance, this novel method fully duplicated the degree of I/R injury and subsequent post-I/R cardiac dysfunction in mice compared with the classic method.

First, efficiency is an advantage of this new model of I/R over the classic approach. The time savings are attributed to the simplified surgical procedure. Compared with the classic method, the chest cavity is opened only once by using a balloon in the new method. The reperfusion operating time in I/R-N was significantly shorter than the classic method. The reperfusion operating time in the I/R-N group was nearly one-eighth of the I/R-C group. At the same time, the total procedure time was also shorter in the I/R-N group than the I/R-C group.

The second advantage of the new model is less tissue damage and inflammation reaction than the classic model of I/R. There are low circulating levels of TNF-α and IL-6 in the new model after the surgery. TNF-α and IL-6 are common pro-inflammatory factors. Tissue damage can elevate TNF-α and IL-6 levels, thus the levels of TNF-α and IL-6 in plasma reflect the degree of tissue damage [33]. Therefore, TNF-α and IL-6 are regarded as measurable indicators of tissue damage. In comparing TNF-α and IL-6 levels between the I/R-N and I/R-C groups, the former group was almost two-thirds of the latter group. This result indicated less tissue damage in the I/R-N group than the I/R-C group, which was due to reduced operation time and invasive surgical procedure in the I/R-N group. Compared with the classic method performed by Hashmi et al, the IL-6 level in our classic method was similar to their values, which indicated our operative skills were comparable with another laboratory [34]. Additionally, the levels of cTnT and LDH were approximate and the degree of neutrophil infiltration in the myocardium was similar between I/R-C group and I/R-N group, which indicated the degree of myocardial damage was no differences between two groups.

Third, an important advantage of the new method was reflected by the recovery time. Mice in the I/R-N and I/R-C groups underwent myocardial I/R injury, hence the time of recovery was longer than the sham group. At the same time, the recovery time in the I/R-N group was significant shorter than the I/R-C group. This result could account for the decreased tissue damage during the surgical procedure because of the difference in times of opening the chest cavity. There was only one mouse dying in the I/R-N group. This result could be due to less tissue damage and a shorter thoracotomy procedure.

Fourth, of greatest importance for the new method was that the differences in extent of myocardial injury and hemodynamic parameters were not statistically significant between the I/R-N and I/R-C groups. The degree of I/R damage and subsequent post-I/R cardiac dysfunction in the new method were fully duplicated compared with the classic method. As the basis of the same injury between the two groups, the new method is more suitable for the murine myocardial I/R model than the classic method.

## Conclusions

Taken together, this study demonstrates that we have developed an efficient surgical procedure for inducing myocardial I/R model in mice. Four main advantages exist in our new method compared with the classic method, as follows: 1. less time-consuming; 2. less tissue damage; 3. shorter recovery time; and 4. identical degree of myocardial I/R injury. Hence, this new method can be applied widely in the research of myocardial I/R injury with the hope that there will be better surgical procedure models in future studies.

## Limitation and Methodological Consideration

Although our research has demonstrated that the new method has great advantages over the classic method to develop mice myocardial I/R model, there are still two limitations in the experiment. One is no used analgesics. Myocardial I/R model is an invasive surgical procedure, therefore, it is important to manage the pain occurred during and after the procedure, and analgesics should be applied to this invasive experiment. However, it is important to be aware that many analgesics have cardioprotective and other effects [35]. Thus, it is appropriate to consider any influences of analgesics on the experiment results.

Another issue is that chest was closed during ischemia in classic method in order to keep consistent with the experimental conditions of new method in our research. According to numerous literatures, both open-chest model [5, 36] and closed-chest model [24, 25] are used widely during ischemia. The result of comparing new method with open-chest model was unknown. However, cardiac function in open-chest model was characterized by larger ejection fraction and stroke volume with a leftward shift in ventricular volume than those of closed-chest model [37]. Thus, open-chest model may increase heart work and cause more myocardial damage compared with closed-chest model.

Based on our experience, the shorter thoracotomy time, the better postoperative recovery. Several reasons can contribute to promote postoperative recovery in closed-chest model. Most important, closed-chest model may be conducive to lung recruitment, which is vital for postoperative recovery. Besides, temperature and humidity of the heart surface are maintained in close-chest model during ischemia. Further, pectoral muscles were injured and a rib retractor was used in some open-chest model, which could cause undesired tissue damage. In our closed-chest model, the pectoral muscles remain intact and no rib retractor was used. This is significant because intact pectoral muscles are essential to cover the wound of intercostal muscles and avoid the suturing of the muscle. Thus, the possibility of surgical complications could be decreased.
